# *Lactobacillus plantarum* ST-III culture supernatant ameliorates alcohol-induced cognitive dysfunction by reducing endoplasmic reticulum stress and oxidative stress

**DOI:** 10.3389/fnins.2022.976358

**Published:** 2022-09-14

**Authors:** Zeping Xu, Jinjing Zhang, Junnan Wu, Shizhuo Yang, Yuying Li, Yuyu Wu, Siyuan Li, Xie Zhang, Wei Zuo, Xiang Lian, Jianjun Lin, Yongsheng Jiang, Longteng Xie, Yanlong Liu, Ping Wang

**Affiliations:** ^1^Department of Pharmacy, Ningbo Medical Center Li Huili Hospital, The Affiliated Hospital of Ningbo University, Ningbo, China; ^2^Department of Pharmacy, Affiliated Cixi Hospital, Wenzhou Medical University, Wenzhou, China; ^3^School of Mental Health, Wenzhou Medical University, Wenzhou, China; ^4^The Affiliated Kangning Hospital, Wenzhou Medical University, Wenzhou, China; ^5^School of Pharmaceutical Sciences, Wenzhou Medical University, Wenzhou, China; ^6^The Affiliated Xiangshan Hospital of Wenzhou Medical University, Ningbo, China

**Keywords:** alcohol, *Lactobacillus plantarum* culture supernatant, cognitive dysfunction, PERK, BDNF

## Abstract

**Background:**

Long-term alcohol exposure is associated with oxidative stress, endoplasmic reticulum (ER) stress, and neuroinflammation, which may impair cognitive function. Probiotics supplements can significantly improve cognitive function in neurodegenerative diseases such as Alzheimer’s disease. Nevertheless, the effect of *Lactobacillus plantarum* ST-III culture supernatant (LP-cs) on alcohol-induced cognitive dysfunction remains unclear.

**Methods:**

A mouse model of cognitive dysfunction was established by intraperitoneal injection of alcohol (2 g/kg body weight) for 28 days. Mice were pre-treated with LP-cs, and cognitive function was evaluated using the Morris water maze test. Hippocampal tissues were collected for biochemical and molecular analysis.

**Results:**

LP-cs significantly ameliorated alcohol-induced decline in learning and memory function and hippocampal morphology changes, neuronal apoptosis, and synaptic dysfunction. A mechanistic study showed that alcohol activated protein kinase R-like endoplasmic reticulum kinase (PERK) signaling and suppressed brain derived neurotrophic factor (BDNF) levels via ER stress in the hippocampus, which LP-cs reversed. Alcohol activated oxidative stress and inflammation responses in the hippocampus, which LP-cs reversed.

**Conclusion:**

LP-cs significantly ameliorated alcohol-induced cognitive dysfunction and cellular stress. LP-cs might serve as an effective treatment for alcohol-induced cognitive dysfunction.

## Introduction

Clinical and experimental models showed that excessive alcohol consumption results in multiple organ injuries, including the brain, lungs, and liver (Morris and Yeligar, 2018). Over 75% of autopsies of chronic alcoholics had severe brain damage, and over 50% of detoxified alcoholics displayed some degree of learning and memory impairment (Vetreno et al., 2011). As we all known, alcohol permeates the blood–brain barrier. Excessive alcohol consumption has been associated with brain damage and impaired cognitive function. Several studies demonstrated that chronic alcohol affects cognitive function through induction of endoplasmic reticulum (ER) stress, neuroinflammation, and oxidative stress (Tiwari and Chopra, 2013; George et al., 2018; King et al., 2020). Alcohol-induced cognitive deficits were associated with reduced levels of the neurotrophin BDNF in humans and rats (Silva-Peña et al., 2019).

The hippocampus is a central memory and learning processing area of the brain’s forebrain’s limbic system and includes regions CA1 and CA3 and the dentate gyrus (DG) (Mira et al., 2019). The hippocampus is responsible for information storage conversion and orientation of long-term memory. It is also related to sensory information processing, emotional control, and perceptual motor skills (Obernier et al., 2002a; Herrera et al., 2003; Wilson et al., 2017). Alcohol exposure affected the development of the central nervous system, especially the hippocampus (Eskay et al., 1995; Obernier et al., 2002b; Khan et al., 2018). Heavy alcohol consumption significantly reduced hippocampal volume (Lee et al., 2016). Hallmarks of chronic alcohol use-mediated brain disease include neuroinflammation and neurodegeneration. Chronic alcohol consumption leads to hippocampal oxidative stress, cell loss, inflammatory cytokine release, and decreased expression of brain derived neurotrophic factor (BDNF), and cognitive function damage (Vetreno et al., 2011; Tiwari and Chopra, 2013; Silva-Peña et al., 2019).

Probiotics are live microorganisms, which render healthy and beneficial effects on animals or humans when administered in adequate amounts (Jäger et al., 2019; Cordaillat-Simmons et al., 2020). Probiotics play a positive role in the recovery of brain function. A long-term probiotic diet reduced abnormal animal behaviors, enhancing recognition and memory capabilities and reducing anxiety (Zang et al., 2019). *Lactobacillus plantarum* is a Gram-positive organism found widely in nature. It is a probiotic in the human gastrointestinal tract that exerts regulating human immune functions, chronic metabolic diseases, and mental and neurological functions (Perdigón et al., 1999; Li et al., 2016; Liu et al., 2016). Several studies have showed that *L. plantarum* slows down the aging process, alleviates age-related cognitive impairment, reduces oxidative stress, inflammation, and apoptosis, which can be used as a functional component to prevent neurodegenerative diseases (Zaydi et al., 2020; Cheon et al., 2021; Lin et al., 2021). *L. plantarum* improves cognitive impairment associated with Alzheimer’s disease by improving neuronal damage and neurotransmitter expression (Song et al., 2021). Several studies found that *Lactobacillus plantarum* culture supernatants (LP-cs) can prevent intestinal oxidative stress, reduce inflammation, and treat infected wounds (Ramos et al., 2010; Paszti-Gere et al., 2013). Recent evidence showed that *L. plantarum* could reduce alcohol-induced neuroinflammation (Shukla et al., 2022). However, the role of *L. plantarum* on alcohol-induced cognitive impairment has not been studied, and the mechanism and active components are still unclear. Therefore, in this study, we investigated the effect of LP-cs on alcohol-induced cognitive dysfunction and its mechanism.

## Materials and methods

*Lactobacillus plantarum* ST-III AB161 (CGMCC 22782) was provided by the Biological Experimental Center of Wenzhou Medical University. LP was activated and passaged three times in MRS medium, inoculated in 100 ml of liquid medium according to 3% inoculation amount of culture medium, cultured at 37°C, 5% CO_2_ for 24 h, centrifuged for 10 min at 4°C at 5,000 r/min. The supernatant was filtered through a 0.22 μm filter to obtain extracellular fluid and then stored at 4°C for use. This procedure yielded the LP-cs from the culture at a concentration of 10^9^ colony-forming units/ml bacterial cells.

### Animal and experimental design

Male ICR mice (about 20 g) were obtained from Vital River Laboratory Animal Technology Co., Ltd., Shanghai, China. The mice were randomly divided into control, alcohol exposure (AE), and alcohol exposure and *L. plantarum* ST-III culture supernatant (AE/LP-cs) groups (*n* = 10–14). Mice were maintained on a 12-h light/12-h dark cycle and under standard temperature and humidity conditions. The LP-cs were mixed with drinking water (1:20) at a ratio ensuring one mouse consumed about 8–9 ml per day. Control group mice and AE group mice had free access to sterile water, and AE/LP-cs groups mice had free access to LP-cs. The AE group was given an intraperitoneal injection of alcohol (2 g/kg body weight, once a day, i.p.) for two consecutive days, with two gaps of 2 days without injections. LP-cs and sterile water were replaced every 2 days. Mice were maintained on treatment for 28 days. After treatment, the Morris water maze test was performed to evaluate mice’s spatial learning and memory abilities. Then they were anesthetized with Avertin. For histomorphological analysis, the animals were perfused with 4% paraformaldehyde in 0.1 M phosphate-buffered saline following the saline solution perfusion. The brains were rapidly detached and post-fixed by immersion in 4% paraformaldehyde. For molecular biological analysis, the hippocampus was separated from the brain after perfusion with 0.9% saline solution and rapidly stored at −80°C. The Laboratory Animal Ethics Committee of Wenzhou Medical University and the Laboratory Animal Center of Wenzhou Medical University approved the animal protocols.

### Morris water maze test

The test was performed in a circular pool with a diameter of 120 cm and a height of 40 cm (Jiliang, Shanghai, China). The pool was filled with opaque water colored with milk powder and maintained at 26 ± 1°C. The training was performed using a hidden circular platform with six blocks of three 60 s trials separated by 20 min inter-block intervals as previously described (Nicholas et al., 2006; Wu et al., 2020a). During the training, the platform remained in the same location relative to the distal cues in the room. For each trial, mice were placed in the water at different start locations (E, S, W, and N) equally spaced and offset from the goal location by 45°. One hour following the sixth block, the hidden platform was removed, and the mice were scored during a 60 s probe trial. They were scored for latency to reach the goal and memory recall, determined by crossing over the previous platform location. Another probe trial was performed 24 h after training to assess memory consolidation and retrieval (Wu et al., 2020b).

### Hematoxylin-eosin and Nissl staining

For hematoxylin-eosin (H&E) staining, 50 μm brain sections were deparaffinized and hydrated, then stained with H&E solution. For Nissl staining, the tissue sections were deparaffinized and hydrated according to the manufacturer’s instructions (Beyotime) and stained with cresyl violet and Nissl differentiation solution. Finally, the hippocampal cell morphology was observed and photographed under a Nikon ECLIPSE 80i microscope (Nikon, Tokyo, Japan).

### Western blot analysis

The hippocampus was homogenized in a lysis buffer containing a protease inhibitor cocktail (10 μL/ml, GE Healthcare Biosciences, PA, United States). The complex was centrifuged at 12,000 rpm/min at 4°C, and supernatants were removed to determine protein concentrations, calculated according to the standard curve. Proteins were separated on 10 or 12% SDS-polyacrylamide gels at 80 V for 2 h. After separation at 300 mA for 1.5 h, the protein bands were transferred to PVDF membranes. The membranes were blocked with 5% skimmed milk for 1.5 h. After washing three times with Tris-buffered saline with 0.05% Tween 20, the membranes were incubated with the primary antibodies overnight: JNK1/2, p-JNK1/2, GAPDH, superoxide dismutase 2 (SOD2), IL-6 (Proteintech, Chicago, IL, United States), Bax, Bcl-2, C-caspase3, postsynaptic dense protein 95 (PSD95), Nrf2, p-CREB133, BDNF, IL-1β, synapsin-1 synaptophysin, GRP78, ATF, protein kinase R-like endoplasmic reticulum kinase (PERK), p-PERK, p-IRE1α, CREB, p-CREB129, PKA, and IL-10 (Abcam, Cambridge, United Kingdom). The following day, membranes were incubated in corresponding secondary antibodies for 1 h at room temperature. Signals were visualized using the ChemiDocXRS + Imaging System (Bio-Rad). Blot bands were quantified using ImageJ software densitometry and expressed as relative density to GAPDH.

### Immunohistochemistry

After dewaxing and hydration, brain sections were incubated in 3% H_2_O_2_ for 15 min, then in blocking solution for 45 min. Subsequently, sections were incubated with primary antibodies at 4°C overnight: SOD2 (Proteintech, Chicago, IL, United States), BDNF (Abcam, Cambridge, United Kingdom). After washing three times in phosphate-buffered saline, the sections were incubated with horseradish peroxidase-conjugated secondary antibodies for 4 h at 37°C. Then, the sections were reacted with 3,3-diaminobenzidine and imaged using a Nikon ECLIPSE 80i microscope (Nikon, Tokyo, Japan).

### Statistical analysis

Experiments were repeated at least three times, and the tissues from each replicate were from different mice. The data results were expressed as mean ± SEM. The experimental data were analyzed using one-way ANOVA and *post hoc* Tukey or Tukey–Kramer tests. All statistical tests were conducted using GraphPad Prism 8.0 (San Diego, CA, United States). The optical density value of Nissl bodies was quantified by ImageJ 6.0 software. Differences with *p* < 0.05 were considered statistically significant. All figures were typeset using PowerPoint (Redmond, WA, United States) or Adobe Photoshop (San Jose, CA, United States) software.

## Results

### *Lactobacillus plantarum* culture supernatants rescues learning and memory impairment induced by alcohol

**FIGURE 1:**
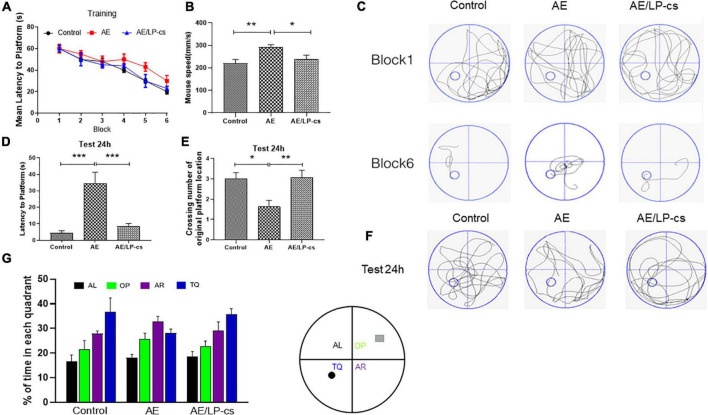
*Lactobacillus plantarum* culture supernatants rescues learning and memory impairment by induced alcohol. **(A)** The learning curve of training period of mice during six blocks in the Morris water maze test. **(B)** Swimming speed of mice in control, AE, and AE/LP-cs mice during Morris water maze test. **(C)** Representative swimming track of mice at blocks one and six during the training period. **(D)** Latency time to find the platform of mice in the probe trial (24 h after training). **(E)** Number of crossing over the original platform location of mice in the probe trial (24 h after training). **(F)** Representative swimming track of mice in the probe trial (24 h after training). **(G)** Percentage of residence time in each quadrant of mice in each group. The quadrant with the platform was designated as TQ, and the quadrant from which the mice started their swimming was designated as OP for “opposite”; the quadrant on the left side of OP was designated as AL for “adjacent left,” and the quadrant on the right side of OP was designated as AR for “adjacent right.” Control vs. AE and AE vs. AE/LP-cs, *p < 0.05, **p < 0.01, ***p < 0.001, *n* = 10–14.

The Morris water maze test reflects mice’s spatial and long-term spatial memory (Bromley-Brits et al., 2011). The time for the AE group to reach the platform from blocks four to six was longer than that of the control and AE/LP-cs groups. After training, the time to reach the platform was significantly reduced for all mice. Furthermore, there was significant difference in swimming speed between AE/LP-cs and AE groups (Figures 1A–C). At 24 h after water maze training, each group’s learning and memory function was tested again (Figures 1C–E). After 24 h, we found that AE mice had fewer crossings over the platform position and had longer latencies to the platform than those in control mice. Compared with the alcohol-induced group, the first time to the platform in the AE/LP-cs group was significantly shorter, and the number of crossings of the platform was significantly more significant (Figures 1D,E). The swimming track during the memory test further suggested that the memory of the AE mice was worse than the control and AE/LP-cs groups (Figure 1F). We also found that mice in the AE group had significantly lower percentages of time in the TQ quadrant than the control and AE/LP-cs groups during trial (Figure 1G). These results suggest that LP-cs improves alcohol-induced decline in learning and memory.

### *Lactobacillus plantarum* culture supernatants ameliorates alcohol-induced changes in hippocampus morphology

**FIGURE 2:**
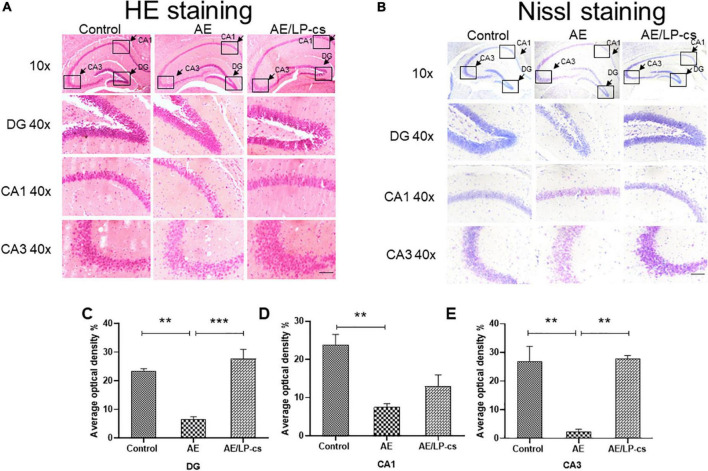
*Lactobacillus plantarum* culture supernatants ameliorates alcohol-induced changes in hippocampus morphology. **(A)** The H&E staining of the hippocampus from mice. **(B)** Nissl staining of the hippocampus from mice (scale bar, 15 μm). **(C–E)** The average optical density value of Nissl bodies was quantified by ImageJ 6.0 software. Control vs. AE and AE vs. AE/LP-cs, *p < 0.05, **p < 0.01, ***p < 0.001, *n* = 3–6.

We investigated whether the hippocampal structure and cellular morphology were altered based on behavioral findings of impaired learning and memory in mice by H&E and Nissl staining (Figure 2). In the control group, the DG, CA1, and CA3 regions of the hippocampus of the mice had normal morphology, the cell layers were arranged tightly, uniformly, and neatly and the staining was uniform. The morphology of DG, CA1, and CA3 in the hippocampus of mice in the AE group changed significantly, including loose, sparse, and disordered cell arrangements, evident cell degeneration, necrosis and shedding, and indistinct cell membranes. The structure of hippocampal neurons in the brain of the AE/LP-cs group tended to be normal, and the pyramidal cells layer were arranged tightly and uniformly, significantly different from the AE group. In addition, we quantified the average optical density values of Nissl bodies in the DG, CA1, and CA3 of hippocampal neurons (Figures 2C–E). Which were significantly lower in the AE group compared with the control group. After administration with LP-cs, the DG and CA3 neurons damage were significantly mitigated as presented with higher average optical density of Nissl bodies. However, there was no significant difference of average optical density of Nissl bodies in the CA1 between the AE and AE/LP-cs group.

### *Lactobacillus plantarum* culture supernatants alleviates alcohol-induced hippocampal apoptosis

**FIGURE 3:**
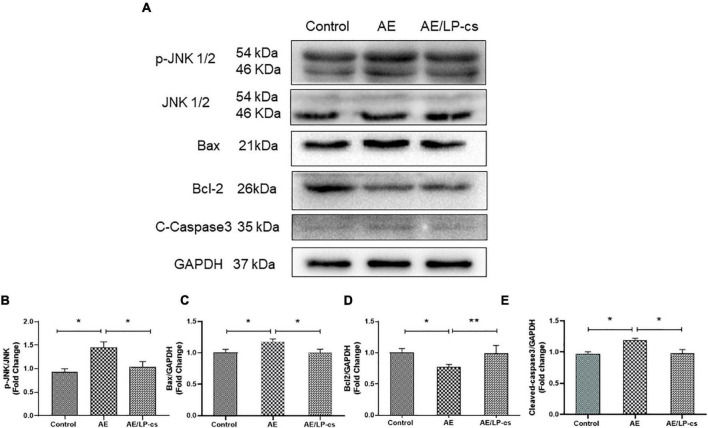
*Lactobacillus plantarum* culture supernatants alleviates alcohol-induced hippocampal apoptosis. **(A–E)** Western blot and quantitative analysis of JNK, p-JNK, Bax, Bcl2, and Cleaved-Caspase3 expressions in the hippocampus from the mice. Control vs. AE and AE vs. AE/LP-cs, *p < 0.05, **p < 0.01, *n* = 5–10.

We measured hippocampal cell apoptosis. C-Jun N-terminal kinase (JNK) is associated with apoptosis and plays an essential role in regulating apoptosis. We measured protein expression levels of JNK and p-JNK in the hippocampus and found that phosphorylation levels of JNK in the AE group were significantly higher than in the other two groups (Figures 3A,B). The expression levels of the pro-apoptotic protein Bax and Cleaved-caspase 3 protein in the AE/LP-cs group were significantly lower than those in the AE group. Expression levels of the anti-apoptotic protein Bcl-2 were significantly higher than in the AE group and were close to the control group (Figures 3A,C–E). These findings suggest that LP-cs significantly alleviates alcohol-induced increases in JNK phosphorylation levels, decreases Bcl-2 expression, and upregulates Bax expression. All results indicates that alcohol causes hippocampal cell apoptosis, and LP-cs protects hippocampal cell apoptosis induced by alcohol.

### *Lactobacillus plantarum* culture supernatants relieves alcohol-induced downregulation of synapse-related proteins

**FIGURE 4:**
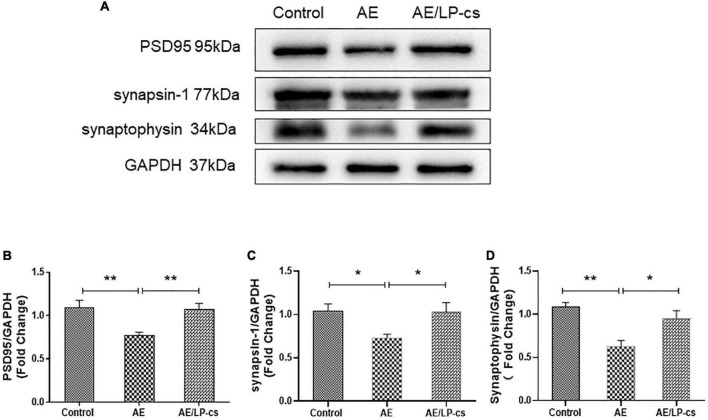
*Lactobacillus plantarum* culture supernatants relieves alcohol-induced downregulation of synapse-related proteins. **(A–D)** Western blot and quantitative analysis of PSD95, synaptophysin, and synapsin-1 expressions in the hippocampus from the mice. Control vs. AE and AE vs. AE/LP-cs, *p < 0.05, **p < 0.01, *n* = 5–10.

Synapses are specialized regions of the contact portion of neurons specifically labeled with synaptic proteins. PSD95 is located beneath the postsynaptic membrane, is pressure-sensitive, and enhances synaptic remodeling. Synapsin-1 and synaptophysin promoted the regulated release of neurotransmitters, which is conducive to the recovery of nerve function (Kacířová et al., 2021; Patzke et al., 2021). These synapse-related proteins can be used to reflect hippocampal function. Western blot results showed that PSD95 and synapsin-1 in the AE group were lower compared with the control group. The expression level of PSD95, synapsin-1, and synaptophysin in the AE/LP-cs group was more robust than that in the AE group and was similar to the control group (Figures 4A–D). These results suggest that alcohol reduces the expression of synapse-related proteins, and LP-cs improves it.

### *Lactobacillus plantarum* culture supernatants alleviates alcohol-induced ER and oxidative stress in the hippocampus

**FIGURE 5:**
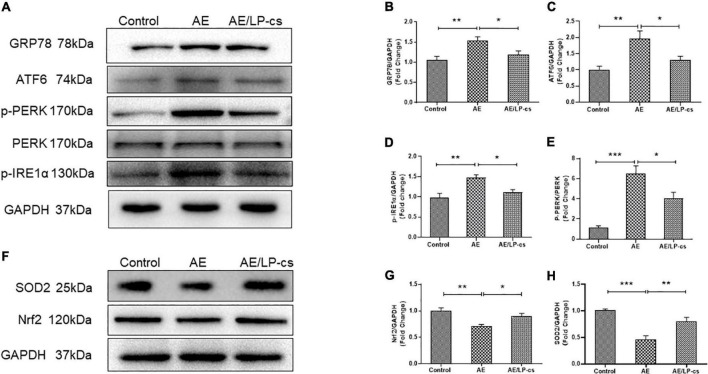
*Lactobacillus plantarum* culture supernatants alleviates alcohol-induced elevated ER stress and oxidative stress in the hippocampus. **(A–E)** Western blot and quantitative analysis of the ER stress-related protein expression in the hippocampus from the mice. **(F–H)** Western blot and quantitative analysis of the Nrf2 and SOD2 expressions in the hippocampus from the mice. Control vs. AE and AE vs. AE/LP-cs, *p < 0.05, **p < 0.01, ***p < 0.001, *n* = 5–10.

Endoplasmic reticulum stress is a process in which the protein folding function of the ER becomes disordered when subjected to endogenous or exogenous stimuli. Many unfolded or misfolded proteins accumulate in the ER cavity and cause subsequent reactions such as induction of GRP78 expression (Oakes and Papa, 2015; Ibrahim et al., 2019). The ER stress sensor pathways, including IRE1/sXBP1, PERK/EIf2, and ATF6, orchestrated the primary regulatory circuits to ensure ER homeostasis (Di Conza and Ho, 2020). We analyzed these pathway-related proteins and found that expression level of GRP78 and ATF6 in the AE group was higher than that in the control group, and these proteins in the AE/LP-cs group were significantly lower than that in the AE group. We also found that phosphorylation levels of IRE1α and PERK were significantly higher in the AE group than in control and AE/LP-cs groups (Figures 5A–E). These findings suggest that alcohol induces ER stress and LP-cs alleviate alcohol-induced ER stress in the hippocampus.

Alcohol causes oxidative stress in the hippocampus (Zhao et al., 2021). When the antioxidant defense system is turned on, Nrf2 activates the transcription of antioxidant genes such as SOD2 (Hoang et al., 2019; Durymanov et al., 2020). Our western blot findings suggested that protein expression level of Nrf2 and SOD2 in the AE group was significantly lower than that in the control group, and protein expression level of Nrf2 and SOD2 in the AE/LP-cs group was close to that in the control group (Figures 5F–H). These findings suggest that alcohol reduces the antioxidant capacity of the hippocampus while LP-cs improves its antioxidant capacity.

### *Lactobacillus plantarum* culture supernatants ameliorates alcohol-induced suppression of CREB activity and brain derived neurotrophic factor expression

**FIGURE 6:**
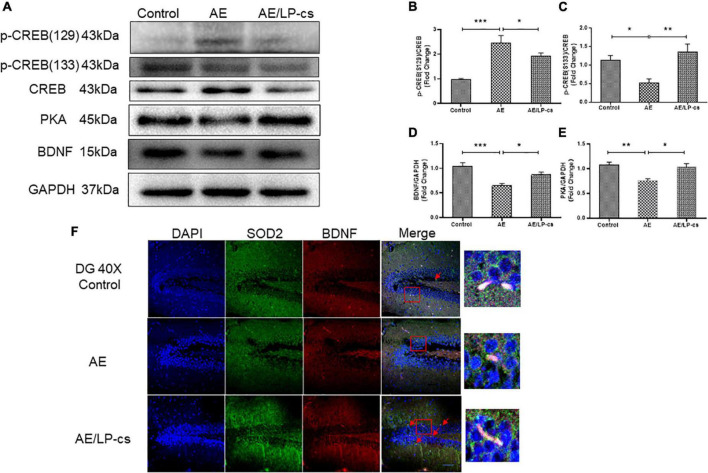
*Lactobacillus plantarum* culture supernatants ameliorates alcohol-induced suppression of CREB activity and BDNF expression. **(A–E)** Western blot and quantitative analysis of BDNF, PKA, p-CREB (S133), and p-CREB (S129), *n* = 5–10. **(F)** Co-immunofluorescence staining of BDNF and SOD2 in the DG of the hippocampus (scale bar, 15 μm, *n* = 3–6). Control vs. AE and AE vs. AE/LP-cs, *p < 0.05, **p < 0.01, ***p < 0.001.

CREB is essential for long-term synaptic plasticity changes, and synaptic plasticity mediates the conversion of short-term memory to long-term memory (Saura and Valero, 2011). Ser133 of the N-terminal kinase-inducible domain of CREB is the phosphorylation site of PKA, and p-CREB is the initiating factor for the expression of downstream gene BNDF, which affects synaptic transmission (Jagasia et al., 2009; Wu et al., 2020a). BDNF promotes the repair and survival of nerve cells, reduces cell apoptosis, and improves learning and memory (Michalski and Fahnestock, 2003; Lu et al., 2014). In the present study, phosphorylation levels of Ser133 protein of CREB in the AE group were significantly higher than those of Ser129 in the other two groups. Protein expression level of PKA and BNDF in the AE group was significantly lower than that in control and AE/LP-cs groups (Figures 6A–F). Consistent with western blot results, co-immunofluorescence staining showed that the fluorescence intensities of BDNF and SOD2 in the AE group were significantly lower than those in the control group, consistent with western blot results (Figure 6F). The results suggest that alcohol induces suppression of CREB activity and BDNF expression in the hippocampus, affecting learning and memory function, and LP-cs reverses these effects.

### *Lactobacillus plantarum* culture supernatants alleviates alcohol-induced inflammation

**FIGURE 7:**
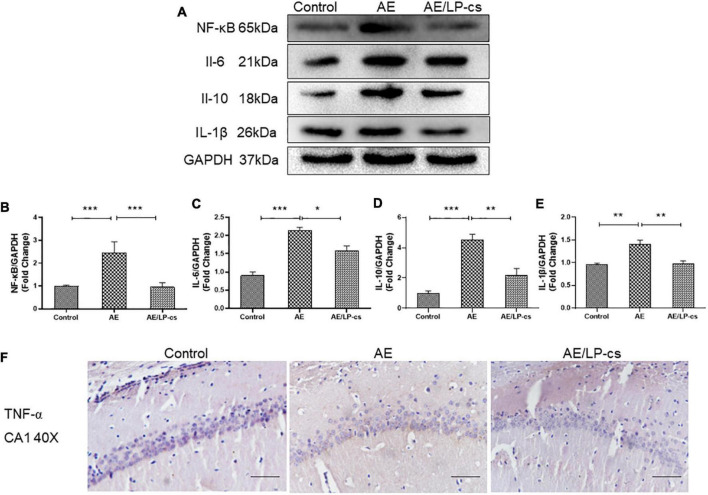
*Lactobacillus plantarum* culture supernatants alleviates alcohol-induced inflammation. **(A–E)** Western blot and quantitative analysis of NF-κB, IL-1β, IL-6, and IL-10, *n* = 5–10. **(F)** The immunohistochemical staining of TNF-α in the CA1 of the hippocampus (scale bar, 15 μm, *n* = 3–6). Control vs. AE and AE vs. AE/LP-cs, *p < 0.05, **p < 0.01, ***p < 0.001.

The NF-κB pathway is a canonical pro-inflammatory signaling pathway based on the role of NF-κB in pro-inflammatory gene expression (Lawrence, 2009). Studies showed that alcohol induces increased expression of inflammatory factors, including NF-κB, in the hippocampus (Wang et al., 2020). In the present study, expression level of NF-κB, IL-1β, IL-6, and IL-10 in the AE group were higher than that in control and AE/LP-cs groups. The expression level of NF-κB, IL-1β, IL-6, and IL-10 in the/LP-cs group were similar to that in the control group (Figures 7A–E). Moreover, we found that TNF-α was deposited in the CA1 region of the hippocampus from the AE group compared with the control group and was partially reversed by LP-cs administration (Figure 7F). These results suggest that LP-cs reduces alcohol-induced inflammation.

## Discussion

We investigated the effect of LP-cs on alcohol-induced cognitive dysfunction. Several studies showed that heavy drinking could damage or lose brain neurons. In the current study, we used a mouse model of alcohol-induced cognitive dysfunction and found that alcohol caused the cognitive decline and reduced spatial learning and memory capabilities. We also found that alcohol can destroy the structure of hippocampal neurons and cause neuronal apoptosis. Treatment with LP-cs relieved alcohol-induced cognitive dysfunction by inhibiting ER and oxidative stress, increasing expression level of BDNF, and improving neuroplasticity.

The hippocampus is a central memory and learning processing area of the forebrain in the limbic system (Mira et al., 2019). One recent study showed that alcohol harmed hippocampal nerves and reduces hippocampal neurons (Dhanabalan et al., 2018). Chronic excessive drinking throughout adulthood and adolescent intermittent ethanol exposure led to the loss of the cholinergic neuronal phenotype within the basal forebrain, reduced hippocampal neurogenesis, and alterations in the frontal cortex (Nunes et al., 2019). Consistent with these studies, we found that alcohol significantly impaired mice’s memory, cognitive function, and hippocampal formation. In the present study, alcohol induced declines in learning and memory function (Figure 1). Based on behavioral changes in mice exposed to alcohol, we measured changes in the hippocampus. The cells in various hippocampus regions in the AE group’s brain were ranked as disordered, denatured, and necrosed. Several studies found that *L. plantarum* protects nerves in mice. Huang et al. (2021) found that *L. plantarum* PS128 prevented cognitive dysfunction in an Alzheimer’s disease model in mice. Cheon et al. (2021) found that heat-killed *L. plantarum* 200655 isolated from Kimchi had neuroprotective effects. Similar to these findings, we found that LP-cs protected against alcohol-induced cognitive impairment. Alcohol significantly increased hippocampal neuronal apoptosis compared with control mice, while LP-cs significantly prevented these changes induced by alcohol. Alcohol significantly increased expression levels of the pro-apoptotic protein Bax, activated caspase 3 protein, and the phosphorylation level of JNK and decreased the expression levels of anti-apoptotic protein Bcl-2; LP-cs reversed these changes (Figure 3). These results suggest that LP-cs prevents hippocampal injury and improves learning and memory functions in alcohol-exposed mice.

Alcohol can induce ER stress. When a cell’s need for protein synthesis exceeds the ER’s capacity to ensure accurate protein folding, an unfolded protein response is initiated. In such cases, the accumulation of misfolded or unfolded proteins, known as ER stress, is sensed via three ER transmembrane proteins (IRE1α, PERK, and ATF6), and the unfolded protein response is activated, with the goal of re-establishing normal ER function (Walter and Ron, 2011). ER stress can promote alcohol-induced neurodegeneration. Blocking ER stress reduced alcohol-induced neurodegeneration (Wang et al., 2021). Previous studies reported that probiotics could reduce ER stress in intestine and liver. Ferrari et al. (2021) found that probiotic supplements reduced ER stress associated with gliadin intake in a mouse model of gluten sensitivity. Liu et al. (2018) found that a novel selenium-glutathione-enriched probiotics attenuated liver fibrosis by activating ER stress. In the present study, we further found that LP-cs treatment could reduce brain ER stress and improve alcohol-induced cognitive dysfunction. As was shown that alcohol significantly increased expression level of GRP78 and ATF6 and phosphorylation levels of IRE1α and PERK, however, LP-cs reversed these changes (Figures 5A–E). These results suggest that LP-cs improves alcohol-induced ER stress. PERK is one of the essential transmembrane proteins in the ER. PERK signal plays a vital role in neuronal apoptosis (Wu et al., 2021). PERK signaling also inhibits the expression of BDNF by phosphorylation of CREB at S129 and PSD95, which subsequently affects the stability of dendrites and mediates the decline of memory after traumatic brain injury (Sen et al., 2017; Sharma et al., 2018). BDNF can regulate neurogenesis by increasing the proliferation of neural stem cells, promoting the differentiation and maturation of the hippocampus, and the development and formation of brain circuits (Scharfman et al., 2005; Waterhouse et al., 2012; Park and Poo, 2013; Vithlani et al., 2013). Therefore, we speculated that PERK signaling might be involved in the regulation of the process of alcohol-induced cognitive dysfunction. In this study, AE increased phosphorylation of Ser129 protein of CREB and decreased expression levels of BDNF and LP-cs reversed these changes (Figures 6A,B,D). In conclusion, our findings suggest that LP-cs improves the cognitive function of mice by inhibiting ER stress, regulating the PERK pathway, and increasing expression levels of BDNF.

Oxidative stress results from an imbalance in the redox system, and alcohol can cause oxidative stress (Kang et al., 2021; Lopatynska-Mazurek et al., 2021; Rumgay et al., 2021). Nrf2 plays an important role in antioxidation by reducing oxidative stress and inflammation and protecting cells by regulating antioxidant enzyme activities through the antioxidant damage pathway. SOD2 is an endogenous antioxidant enzyme synthesized in the cytoplasm and translocated to the mitochondria. SOD2 is also critical for defense against oxidative stress. In the present study, we found that alcohol reduced the expression level of Nrf2 and SOD2 in mice, causing an imbalance in the redox system, while LP-cs reversed these effects (Figures 5F–H). Similar to our findings, a study found that *L. plantarum* ZS62 pretreatment appeared to confer hepatic protection on alcohol-induced subacute hepatic damage by enhancing antioxidative capacity (Gan et al., 2021). In addition, oxidative stress is related to macromolecule damage and activation of several cell survival and death pathways. The signal pathways involved in this effect include NF-κB (Paithankar et al., 2021). Intracellular ROS can activate cellular NF-κB (Kratsovnik et al., 2005). In the brain, NF-κB regulates synaptic plasticity and memory, neuroinflammation, and neuron survival; it plays a vital role in neuroplasticity, nerve inflammation, and neurodegeneration associated with alcohol dependence and abuse (Russo et al., 2009). Alcohol imbalances the redox system, leading to activation of NF-κB; it is accompanied by an increase in the expression of inflammatory factors (i.e., IL-1β, IL-6, IL-10, and TNF-α) and a decrease in the expression of synapse-related proteins including PSD95, synapsin-1, and synaptophysin. LP-cs can protect neurons by inhibiting NF-κB signaling (Figures 4, 7). These findings suggest that the downregulation of the NF-κB system appears to be an adaptive mechanism that protects the remaining neurons from alcohol neurotoxicity and neuroinflammation. LP-cs can protect neurons by inhibiting oxidative stress, NF-κB signaling pathway, inflammation, and improving synaptic plasticity.

## Conclusion

**FIGURE 8:**
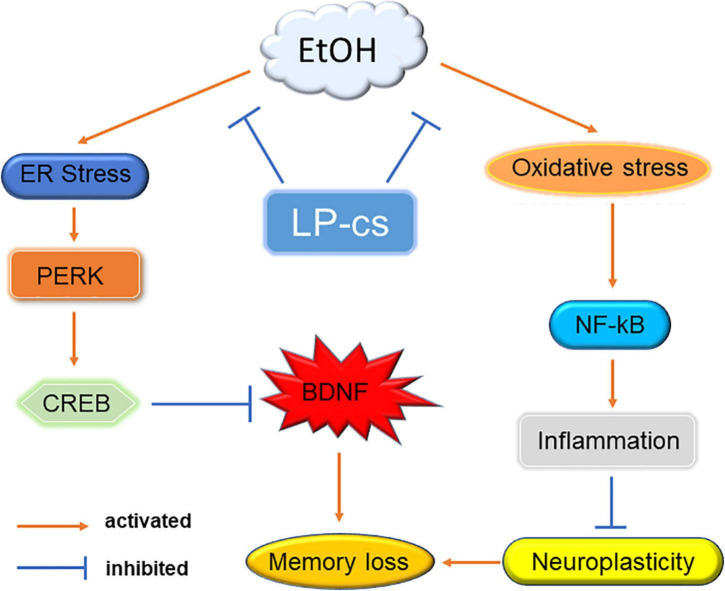
Mechanism. Alcohol-induced ER stress activates PERK signaling to regulate CREB expression, suppressing BDNF levels. Alcohol induces oxidative stress to activate NF-κB expression and trigger an inflammatory response, affecting synaptic plasticity. Alcohol eventually leads to memory impairment through these two pathways. LP-cs alleviates alcohol-induced cellular stress.

Alcohol induces cognitive dysfunction. Mechanistic studies showed that alcohol causes oxidative and ER stress, causing PERK and NF-κB signal transduction in the hippocampus, triggering neuronal apoptosis, affecting neuronal function, and finally leading to memory loss. LP-cs prevent alcohol-induced cellular stress and inhibit PERK signaling from increasing the expression of BDNF. It also inhibits NF-κB signaling to regulate the expression of inflammatory factors and improve neuroplasticity, thereby improving cognitive dysfunction caused by alcohol (Figure 8). Our results suggest that LP-cs might treat cognitive impairment or disorders caused by alcohol. With the increasing use of alcohol, it is necessary to study the mechanism of alcohol-induced cognitive dysfunction to provide a basis for its treatment. A significant contribution of this study is that it further elucidates the mechanism of alcohol-induced cognitive dysfunction.

## Data availability statement

The original contributions presented in this study are included in the article/supplementary material, further inquiries can be directed to the corresponding authors.

## Ethics statement

The animal study was reviewed and approved by the Laboratory Animal Ethics Committee of Wenzhou Medical University and the Laboratory Animal Centre of Wenzhou Medical University.

## Author contributions

YLL, PW, and LTX provided the idea for the study. YLL, JZ, and ZX contributed to the study design. ZX, PW, and JW gave important and thoughtful advices and performed the experiments. SY, YYL, YW, SL, XZ, LTX, JL, and YJ provided assistance with the experiments. All authors approved the final manuscript for submission.
